# Nanoemulsions of *Phoenix dactylifera* L. (Decaffeinated) and *Coffea arabica* L. Extracts as a Novel Approach for the Treatment of Carbon Tetrachloride-Mediated Liver Fibrosis

**DOI:** 10.3390/antiox13030355

**Published:** 2024-03-16

**Authors:** Eman S. Alamri, Hala M. Bayomy, Mohamed A. Mohamady Hussein, Nawal A. Ozaybi, Seham E. Almasoudi, Nahla S. Zidan, Renad A. Albalwi, Hebatallah H. Atteia, Fayza M. EL-Ezaly

**Affiliations:** 1 Food Science and Nutrition Department, Faculty of Science, University of Tabuk, Tabuk 71491, Saudi Arabia; 2 Department of Pharmacology, Medical Research and Clinical Studies Institute, National Research Centre, Dokki, Giza 12622, Egypt; 3 Department of Nutrition and Food Science, University of Tabuk, Tabuk 71491, Saudi Arabia; 4 Department of Pharmaceutical Chemistry, Faculty of Pharmacy, University of Tabuk, Tabuk 71491, Saudi Arabia; 5 Department of Home Economics, Faculty of Specific Education, Mansoura University, Mansoura 35516, Egypt

**Keywords:** liver fibrosis, nanoemulsions, decaffeinated palm date seeds, Arabic coffee seeds

## Abstract

Liver fibrosis is a condition characterized by the excessive buildup of scar tissue in the liver. This scarring occurs as a result of chronic liver damage, often caused by conditions such as hepatitis, alcohol abuse, certain metabolic disorders, genetic abnormalities, autoimmunity, and noninfectious diseases such as fatty liver which leads to liver fibrosis. Nanoparticles have gained attention in recent years as potential therapeutic agents for liver fibrosis. They offer unique advantages due to their small size, large surface area, and ability to carry drugs or target specific cells or tissues. Studies have suggested that nanoemulsions may enhance drug delivery systems, enabling targeted drug delivery to specific sites in the liver and improving therapeutic outcomes. In this study, we explore the protective and therapeutic values with phytochemical profiling of the used agro-wastes decaffeinated palm date seeds (*Phoenix dactylifera* L., PSC) coffee and caffeinated Arabic coffee seeds (*Coffea arabica* L.; ACS). Both ACS and PSC extracts were converted into nanoemulsion (NE) forms using the oleic acid/Tween 80 system, which was recruited for the purpose of treating a rat model with liver fibrosis. Transmission electron microscopy (TEM) and dynamic light scattering (DLS) were used to record the sizes, morphologies, hydrodynamic diameters, and ζ-potentials of the prepared NE-ACSE and NE-PSCE. Accordingly, the NE-ACSE and NE-PSCE imaged via TEM and their ζ-potentials were recorded at 20.7, 23.3 nm and −41.4, −28.0 mV, respectively. The antioxidant properties were determined with a DPPH scavenging assay. The synthesized NE-PSCE and NE-ACSE were employed to treat a rat model with CCl_4_-induced liver fibrosis, to estimate the role of each emulsion-based extract in the treatment of liver fibrosis through recording inflammatory parameters, liver functions, antioxidant enzymes, and histopathological analysis results. The nanoemulsion forms of both ACSE and PSCE provided significant increases in antioxidant enzymes, reducing inflammatory parameters, compared to other groups, where liver functions were decreased with values close to those of the control group. In conclusion, both nanoemulsions, ACSE and PSCE, provided a new avenue as therapeutic approaches for liver diseases, and further studies are encouraged to obtain maximum efficiency of treatment via the combination of both extracts.

## 1. Introduction

Chronic liver disease encompasses a wide range of conditions, including non-alcoholic fatty liver disease (NAFLD), hepatitis B and C, cirrhosis, and autoimmune liver diseases [1]. In liver fibrosis, fibrous scar tissues are accumulated in the liver with an excessive pattern [2]. This scarring occurs as a result of chronic liver damage, often caused by conditions such as hepatitis, alcohol abuse, certain metabolic disorders, genetic abnormalities, autoimmunity, and noninfectious diseases such as fatty liver which leads to liver fibrosis [3]. In advanced liver fibrosis, impaired blood flow can cause fluid to accumulate in the abdominal cavity, resulting in a condition called ascites [2]. Liver fibrosis can impair the liver’s ability to remove toxins from the bloodstream, leading to hepatic encephalopathy, a condition characterized by confusion, cognitive impairment, and even coma [4,5]. Prolonged liver fibrosis increases the risk of hepatocellular carcinoma [6].

Nanoparticles have gained attention in recent years as potential therapeutic agents for liver fibrosis. They provide unique properties due to their small size, large surface area, and ability to carry drugs or target specific cells or tissues. Several types of nanoparticles have been investigated for the treatment tof CCl4-induced liver fibrosis. These include liposomes [7], and polymeric [8], gold [9], and magnetic nanoparticles [10]. For example, liposomal formulations containing antioxidants like vitamin E, N-acetylcysteine, or curcumin have shown promising results in animal models of CCl4-induced liver fibrosis [11].

It is worth mentioning that nanotechnology and nanoemulsions are a rapidly evolving field of research, and there is ongoing exploration of their potential applications in liver disease treatment. Emulsions are colloidal systems comprising dispersed oil droplets in an aqueous bulk phase. This system needs to be stabilized via a surfactant/emulsifier and a co-surfactant [12]. Various techniques such as homogenization and ultrasonication were used to disperse the oleic phase into the aqueous phase, which can help to provide oil droplets in nanoscale in the range of 10 nm to microscale. In nanoemulsions, both the continuous aqueous phase and the dispersed oleic phase are immiscible enough with droplet sizes in the scale of 10 to 500 nm [13,14]. Studies have suggested that nanoemulsions may enhance drug delivery systems, enabling targeted drug delivery to specific sites in the liver and improving therapeutic outcomes [15,16].

However, further research and clinical trials are needed to understand their safety, efficacy, and potential benefits in the treatment of chronic liver disease. The treatment of chronic liver disease depends on the specific underlying cause and severity of the condition. One of the notable aspects of using nanoemulsions is their ability to encapsulate and deliver natural components effectively. These natural components can include bioactive compounds obtained from plants, such as polyphenols, flavonoids, essential oils, and many therapeutic molecules. By encapsulating these components in nanoemulsions, their stability, solubility, and bioavailability can be greatly enhanced. While nanoemulsions of natural components may have some potential in the field of liver disease treatment, it is important to note that they are still under investigation and not yet widely used in clinical practice.

Oleic acid (OA), a naturally occurring fatty acid, is derived from plant and animal sources. OA nanoemulsions are produced through using high-pressure homogenization technology [17]. OA exhibits an inefficient packaging effect in the bulk phase due to a double bond in the cis configuration in the middle of its 18-carbon chain. Thus, OA is very fluid and not viscous or dense at room temperature. This results in the generation of liquid droplets as cores in nanosystems [18]. Thus, using Tween 80 as a surfactant in addition to polyethylene glycol (PEG) as a co-surfactant provides a perfect complement for achieving the NE structure. 

Phoenix dactylifera is sub-classified under the family of Arecaceae, which is known as Palmaceae, and it contains the date seed [19]. Palm date seed coffee has gained popularity in recent years, primarily for its potential health benefits. While more studies are still required to fully understand the extent of such health values, preliminary studies suggest several positive effects of consuming palm date seed coffee due to its antioxidant, anti-inflammatory, and digestive-health-promoting properties, nutritional value, and energy-boosting properties [20,21]. Palm date seed coffee contains phenolic compounds and flavonoids, which exhibit potent antioxidant activity [22,23]. Palm date seed coffee contains dietary fiber, which is important for maintaining a healthy digestive system. Furthermore, coffee with palm date seeds is a good source of important nutrients [24]. It has significant concentrations of iron, calcium, magnesium, potassium, and other elements that are necessary to sustain physiological processes [25].

The Arabian coffee (Coffea arabica) plant is a member of the Coffea genus, which belongs to the family Rubiaceae. Coffee has been investigated for its protective effect against many chronic diseases, such as cancer [26], liver diseases, Parkinson’s disease [27], and diabetes mellitus T2. Regular consumption of coffee can help maintain liver function activity, with a reduction in the risk of chronic liver disease [28,29]. Furthermore, coffee phytochemicals are claimed to improve mitochondrial function and oxidation of lipids, and minimize steatosis risk in liver tissues [30].

Inedible parts of vegetables and fruits are usually thrown away as wastes. However, such agro-wastes contain bioactive compounds with health values, such as phenolic compounds, which display antioxidant properties [22]. In this study, we explore the protective and therapeutic values with phytochemical profiling of the used agro-wastes palm date seed coffee (PSC) and Arabic coffee seeds (ACS). Both ACS extract (ACSE) and PSC extract (PSCE) were converted into nanoemulsion (NE) forms (NE-PSCE, NE-ACSE), and the antioxidant properties were analyzed. An in vivo study was carried out in which the NEs of both ACSE and PSCE were given to animal models with chronic liver disease. In the current study, a green nano-delivery system was fabricated for the efficient delivery of PSCE and ACSE, which were recruited for the purpose of treating a mouse model of liver fibrosis. The composite systems composed of ACSE or PSCE were emulsified into the oil phase of oleic acid. The synthesized NE-PSCE and NE-ACSE were employed to treat a CCl4-induced liver fibrosis model to estimate the role of each emulsion-based extract in the treatment of liver fibrosis through recording inflammatory parameters, liver functions, and antioxidant enzymes.

## 2. Materials and Methods

### 2.1. Materials

The date palm (*Phoenix dactylifera* L.) and Arabica seed coffee (*Coffea arabica* L.) used in this study were purchased from a local market, Malaz Dist., Riyadh, Riyadh Province, Saudi Arabia. Oleic acid, Tween 80, and polyethylene glycol-2000 (PEG-2000) were purchased from Sisco Research Laboratories Pvt. Ltd., Mumbai, India. Carbon tetra chloride (CCL4) of 100% concentration and olive oil were purchased from the Algomhoria Company, Cairo, Egypt.

#### Animals

Healthy male rats (body weight = 190 ± 10 gm) were used in this study. Rats were divided into 6 groups, where each group contained 6 rats. The animals were purchased from the Agricultural Research Center, Giza, Egypt. The experiments were conducted for 2 months and rats were housed in suitable conditions, on a regular light/dark cycle and fed on a basal diet, and the animals were provided with ad libitum access to water for the pharmacokinetic experiments.

### 2.2. Extraction of Date Palm Seed Coffee (PSC) and Arabica Seed Coffee (ACS)

Palm date seeds were taken out and carefully washed in water. At room temperature, the gathered seeds were allowed to dry for two to three days. The dried hard seeds were then ground into a coarse powder using a coffee seed grinder. Coffee seeds were ground as well. Ethanol extracts of PSC and ACS were obtained using the following method. Around 250 g of PSC or ACS was soaked in 10 volumes of ethanol with occasional stirring for 48 h. The macerates were separated by the filtration technique. Then, the filtrate was placed in a rotary evaporator at 50 °C to obtain a condensed extract. The raw extracts were obtained, placed in darkened bottles, and stocked in a deep freezer until utilization.

### 2.3. Gas Chromatography–Mass Spectrometry (GC-MS) Analysis

Using a direct capillary column TG–5MS (30 m × 0.25 mm × 0.25 µm film thickness), a GC-TSQ mass spectrometer (Thermo Scientific, Austin, TX, USA) was used to analyze the chemical composition of the samples. The temperature of the column oven was first maintained at 60 °C, then raised by 5 °C/min to 250 °C and held for 2 min, then raised to 280 °C at a rate of 25 °C/min. At 270 °C, the injector temperature was maintained. As a carrier gas, helium was employed at a steady flow rate of 1 mL/min. Diluted samples containing 1 µL were automatically injected using an Autosampler AS3000 connected to a GC in split mode, with a 4 min solvent delay. In full scan mode, EI mass spectra were obtained at 70 eV ionization voltages covering *m*/*z* 50–650. The ion source and transfer line were respectively set at a temperature of 200 °C. By comparing the mass spectra of the constituent parts with those of the NIST14 and WILEY 09 mass spectral databases, the components were identified.

### 2.4. Preparation of PSCE Nanoemulsions (NE-PSCE) and ACSE Nanoemulsions (NE-ACSE)

NE-PSCE and NE-ACSE were prepared using a water/oil nanoemulsion with some modifications [31,32]. An aqueous phase was achieved by adding Tween 80 (5.5%) to an aqueous solution (87.5%) with seed extract (2.5%), which was then stirred for 30 min at room temperature. The oil phase, containing oleic acid (~4.45%) with PEG (0.05%) as a co-surfactant, was then stirred for 20 min. After complete dissolution, the oil phase was added dropwise into the aqueous phase and stirred for 30 min. Finally, the total emulsion was ultrasonicated for 30 min using a 24 kHz 400 W ultrasonicator (Model UP400S, Ultrasound Technology, Teltow, Berlin, Germany).

### 2.5. Characterization of Nanoemulsions

The particle shapes of NE-PSCE and NE-ACSE were investigated using a transmission electron microscope (TEM; JEM model 1400, 100 kV). The particle size distributions and ζ–potentials of both NE-ACSE and NE-PSCE were recorded using dynamic light scattering (DLS) measurements (Malvern Instruments Ltd., Malvern, UK). DLS analyses were performed at 633 nm. Scattering intensity was recorded using a photodiode detector at a 173° angle relative to backscattering. Each sample was measured 3 times, in 10 runs for each measurement.

### 2.6. Antioxidant Activity Using DPPH Radical Scavenging Assay

In the DPPH assay, the free radical DPPH (DPPH^•^) reacts with antioxidants to provide 2,2-diphenyl-l-picryl hydrazine, which is colorless, where the more colorless it is, the greater its antioxidant properties. Various concentrations of extracts prepared in ethanol were mixed with a determined amount of freshly prepared DPPH^•^ in ethanol as described previously, with some modifications [33]. The prepared mixture was vortexed and then incubated at room temperature in the dark for 0.5 h. The absorbance of the mixtures was recorded at 517 nm using a spectrophotometer. The inhibition % was calculated using the following equation [34]:

Inhibition (%) = [(A_control_ − A_sample_)/A_control_] × 100

where A_control_ is the absorbance of the control and A_sample_ is the absorbance of the tested sample. The efficient concentration that is required to obtain 50% antioxidant activity (EC50) was recorded as well.

### 2.7. Experimental Conditions

#### 2.7.1. Experimental Animal Protocol

There were six sets of Wistar albino rats, each containing 6 animals. The animals were fasted for 24 h before being treated with carbon tetrachloride (CCl4). Intraperitoneal (IP) injection of carbon tetrachloride (CCl4), diluted in olive oil, was administered twice a week at a concentration of 0.5 mg/kg body weight of the rat to cause liver fibrosis [35,36]. All animal groups were given CCl4 except the first group (group 1), which was maintained as a normal control and received normal saline at 5 mL/kg orally. Liver fibrosis groups were divided as follows: group 2 was maintained as a positive group left without treatment, group 3 was treated with a diet of ACSE at 100 mg/kg daily, group 4 was treated with a diet of PSCE at 100 mg/kg daily, group 5 was treated with NE-PSCE at 100 mg/kg daily, and group 6 was treated with NE-ACSE at 100 mg/kg daily.

#### 2.7.2. Calculation of Nutritional Parameters

The rats’ daily food intake was tracked, and once a week, their weight was assessed to determine how much weight they had acquired (body weight gain). At the conclusion of the experiment, the body weight gain, body weight gain percentage, and relative organ weight were determined using the following equations:

Body weight gain % = [Final weight (gm) − Initial weight (gm)/Initial weight (gm)] × 100

Relative organs weight = (organ weight (g)/final body weight (g)) × 100

#### 2.7.3. Biochemical and Enzyme Activities

Glutathione peroxidase (GPx) and malondialdehyde (MDA) were measured according to a previous study [37,38]. Hydrogen peroxide (H_2_O_2_) was measured using a H_2_O_2_ assay, and catalase was recorded with a catalase assay with kits from Biodiagnostic, according to Aebi [39]. Reduced glutathione (GSH) was measured according to [40]. SOD and catalase were recorded according to Nishikimi et al. [41], using specific kits in accordance with the manufacturer’s instructions. Interleukin-6 (IL-6) was assessed and quantified using an enzyme-linked immunosorbent assay (ELISA) according to the manufacturer’s instructions, in accordance with the method of [42]. The C-reactive protein level (CRP) was measured using an immunoturbidimetric assay [43]. Rat TNF-α (tumor necrosis factor alpha), was measured by using the manufacturer’s protocol for a Mouse/Rat Dopamine ELISA Assay Kit (No. 438204 (5 plates); BioLegend, Inc., San Diego, CA, USA). Evaluation of liver functions was calculated through utilization of kits for the following analyses: Alanine aminotransferase (ALT) and aspartate aminotransferase (AST) activities were analyzed according to the method of [44], using kits bought from Randox Company (Crumlin, UK). Serum albumin levels (Alb) were determined according to the method of [45], using kits purchased from Diamond (Germany). Serum globulin (Glb) values were detected by subtracting the albumin from the total proteins. Serum total bilirubin (T-Bil) was determined using the enzymatic colorimetric method according to Balistreri WF and Shaw LM (1987), using kits purchased from Diamond (Hildesheim, Germany).

#### 2.7.4. Histopathological Examination of Liver

Tissue specimens from the liver were collected from the rats, and then they were instantly fixed in 10% buffered formaldehyde solution for 24 h. Then, the fixed tissues were dehydrated with ethanol solutions in differing concentrations and inserted into paraffin wax blocks. Then, paraffin blocks were made by cutting them into slices of 4 microns thickness; then, the cut specimens were placed on glass slides and stained with hematoxylin and eosin staining (H&E) to be photographed under a light microscope, according to [46].

### 2.8. Statistical Analysis

The results were analyzed with the software GraphPad Prism 8.0.2. The data are expressed as means ± SD, as statistical significance was evaluated using one-way ANOVA followed by Tukey’s correction (* *p* < 0.0332; ** *p* < 0.0021; *** *p* < 0.0002; **** *p* < 0.0001; versus control).

## 3. Results

### 3.1. Chemical Compositions of Coffea arabica and Phoenix dactylifera Extracts Obtained Using Gas Chromatography–Mass Spectrometry (GC-MS)

In gas chromatography–mass spectrometry (GC-MS), the features of gas–liquid chromatography along with mass spectrometry are combined in a technical method to identify various substances within a tested sample [47]. Lately, GC-MS has become the primary technical platform for characterizing secondary metabolites in plants and organisms that are not plants [48]. According to the GC-MS analysis, thirty-five (35) compounds were identified in ACSE. The active principles, retention times (RTs), molecular formulas, molecular weights (MWs), and concentrations (%) are displayed in Table 1 and Figure S1. The prevailing compounds were 1h-purine-2,6-dione, 3,7-dihydro-1,3,7-trimethyl-(caffeine), and palmitic acid, TMS derivative, as shown in Table 1. A total of 42 compounds were identified in PSCE. Triethylene glycol, palmitic acid, and oleic acid are the active principles that are shown in Table 2 and Figure S2, together with their RTs, MWs, molecular formulas, and concentration percentages. Because of its greater ability to be extracted, ethanol may yield a variety of active ingredients that are involved in a wide range of biological processes. In order to use them for the creation of traditional medicines, more research is required to extract unique active components from the medicinal plants, which could lead to the development of a new treatment for a number of incurable illnesses [49]. The fatty acid profiles of 14 different types of date seeds that were examined using GC-MS were recorded, as the fat contents were located in the range of 5% to 9% (*w*/*w*). A total of 11 fatty acids were recorded, and oleic acid and palmitic acid were found in most varieties [50]. This was found in a previous study as well [51].

### 3.2. Morphological Investigation of NE-ACSE and NE-PSCE

**Figure 1:**
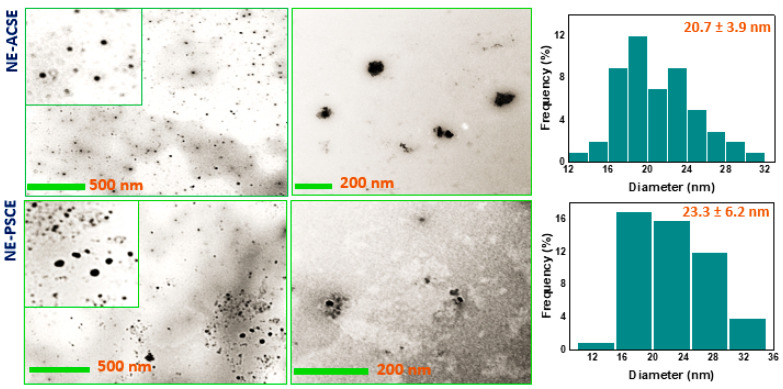
TEM images and particle size distribution histograms of NE-ACSE and NE-PSCE.

The sizes and shapes of the as-prepared NE-ACSE and NE-PSCE were recorded with the help of a transmission electron microscope (TEM), as shown in Figure 1. The images of the NEs recorded with the TEM depict that the prepared NE-ACSE and NE-PSCE are spherical, with nanosizes of 20.7 ± 3.9 nm and 23.3 ± 6.2 nm, respectively.

### 3.3. Distribution of Particle Sizes and ζ-Potentials of Nanoemulsions NE-PSCE and NE-ACSE

**Figure 2:**
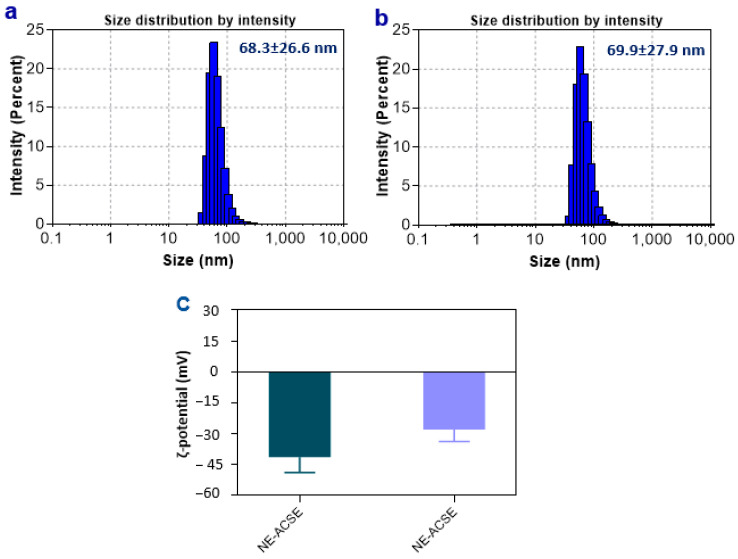
DLS measurements displaying hydrodynamic diameters (particle size distributions) (**a**,**b**) of the developed NE-ACSE and NE-PSCE, respectively. ζ-potentials (**c**) of the developed NE-ACSE and NE-PSCE.

DLS was used to measure the prepared NEs’ particle size distributions and ζ-potentials, as shown in Figure 2. According to the obtained results, NE-ACSE showed a hydrodynamic diameter of 68.3 ± 26.6 nm, whereas NE-PSCE exhibited 69.9 ± 27.9 nm. Moreover, both nanoemulsion samples were imaged as displayed in Figure S3a,b. For the ζ-potentials, as shown in Figure 2c (Figure S4), NE-ACSE displayed −41.4 ± 7.5 mV, whereas NE-PSCE showed −28.0 ± 5.8 mV. The negative charges of the NEs could be attributed to the polyphenols which are available in both ACS and PSC extracts, and also to using Tween 80 (a non-ionic surfactant) and oleic acid as an anionic surfactant in addition to PEG [52]. It was reported that, for nanomaterials with ζ-potentials over ±30 mV, it is necessary to stabilize the NPs in the aqueous suspension [53,54,55]. And it can be observed that NE-ACSE is more stable than NE-PSCE. The particle sizes depicted by the TEM analysis are smaller than in the DLS measurements, as in DLS, nanoparticles could be aggregated to form larger particles. Furthermore, the suspended nanoparticle solution swelled in the solvent used to prepare samples for DLS analysis. The values of the polydispersity index (PDI) were 0.332 and 0.321 (<0.5), which indicates that the NE particles are distributed uniformly when suspended in the solution.

### 3.4. DPPH Scavenging Activity Measurements of Nanoemulsions

**Figure 3:**
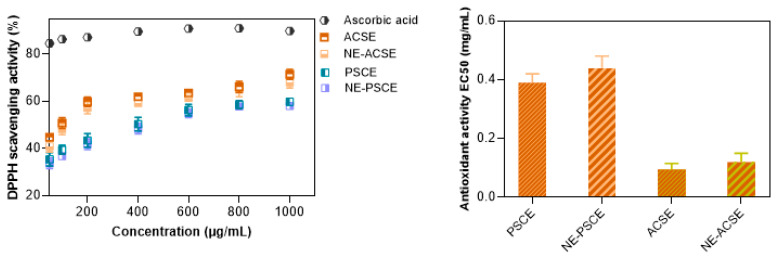
Antioxidant activity of ACS and PSC extracts and their nanoemulsions NE-ACSE and NE-PSCE.

The antioxidant activities of the tested samples were evaluated as shown in Figure 3 using a DPPH radical scavenging assay at various concentrations of the extracts and their nanoemulsions, while recording the efficiency concentration required to obtain a 50% antioxidant effect (EC50). The antioxidant activities of the encapsulated ACSE and PSCE were slightly lower than those of the corresponding free extracts. It is better for bioactive materials to be bio-accessible and released in a sustained manner when displaying the related bioactivity. Though the antioxidant properties of the encapsulated extracts are less than free, the nanoemulsified components are protected against rapid degradation and loss, and their antioxidant activities are maintained efficiently [56]. Such protection could be achieved against rapid pepsin digestion or during storage against light, heat, and oxygen, which encourage the chemical breakdown of the bioactive compounds, lowering their biological value [57,58,59].

Such findings are consistent with a previous study which found that a nanoemulsion of curcumin solid lipid particles provided antioxidant activity using a FRAP assay, with a value of 0.996 ± 0.07 mM L-ascorbic acid equivalent; in addition, the free curcumin displayed antioxidant activity with a value of 2.504 ± 0.06 mM L-ascorbic acid equivalent [60]. The antioxidant properties of ACS extract could be attributed to it being rich in antioxidants including phenolic compounds and flavonoids [61]. The same is true for PSC extract, as its antioxidant activity is a result of high polyphenol content [21].

### 3.5. Effects of NE-PSCE and NE-ACSE on CCL4-Induced Liver Fibrosis in Rat Model

#### 3.5.1. Antioxidant Enzymes and MDA

**Figure 4:**
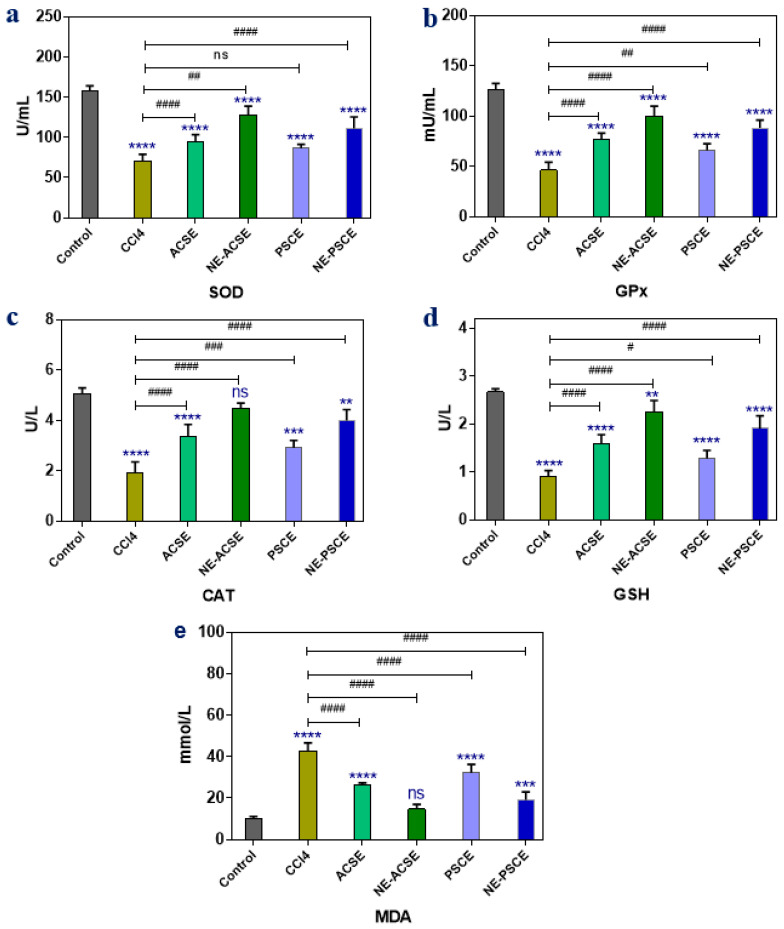
Effects of NE-PSCE and NE-ACSE on oxidative stress biomarkers including antioxidant enzymes and MDA (**a**–**e**) in treated rats with induced liver fibrosis compared to normal and CCl4-induced liver fibrosis groups. Data are expressed as average ± SD (*n* = 6). Where, ns indicates not significant, ** *p* < 0.0021, *** *p* < 0.0002, **** *p* < 0.0001, versus control. ^#^ *p* < 0.0332, ^##^ *p* < 0.0021, ^###^ *p* < 0.0002, ^####^ *p* < 0.0001, versus CCl4 group.

The effects of PSCE, ACSE, and their nanoemulsion forms, NE-PSCE and NE-ACSE, on chronic liver disease-afflicted rats’ antioxidant enzyme and free radical levels, including those of malondialdehyde (MDA), glutathione peroxidase (GPx), catalase (CAT), reduced glutathione (GSH), and superoxide dismutase (SOD), were observed, as illustrated in Figure 4. When compared to the control group, the CCL4 group demonstrated a significant decrease in MDA levels and a significant decrease in the values of SOD, GPX, GSH, and CAT. Treatment with both PSCE and ACSE increased the levels of SOD, GPx, CAT, and GSH while decreasing MDA in comparison to the chronic liver group (CCl4). The administration of the NE-ACSE and NE-PSCE nanoemulsion forms to ill animals resulted in significant improvements in SOD, GPx, GSH, and CAT levels. In this case, NE-ACSE may be able to improve things more than the other treatments.

#### 3.5.2. Liver Functions

**Figure 5:**
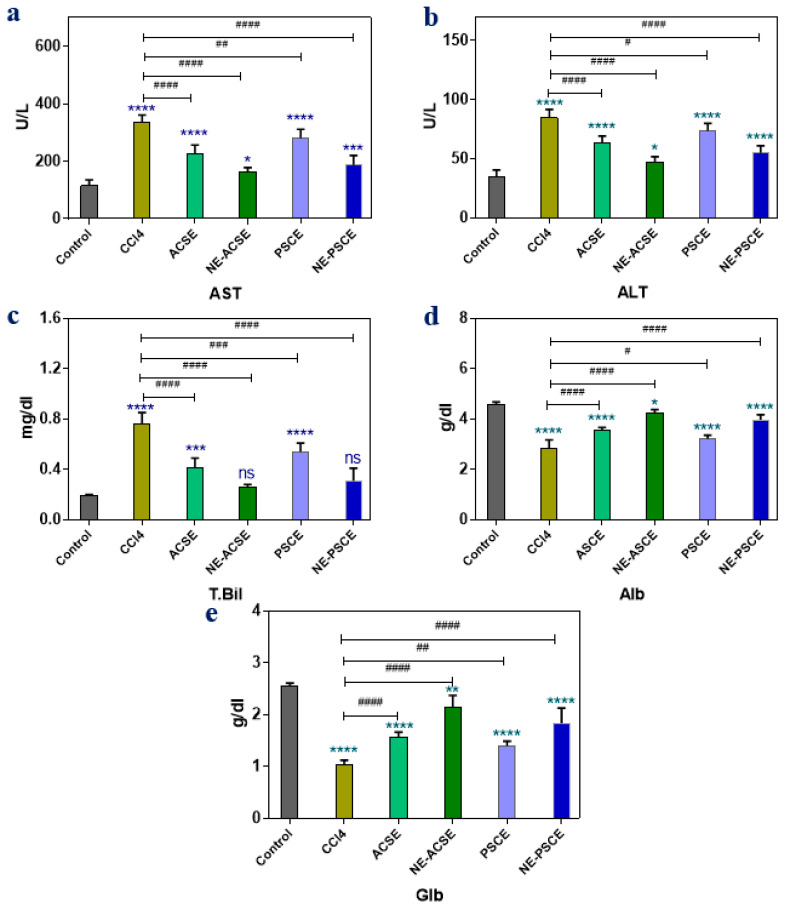
Effects of NE-PSCE and NE-ACSE on liver enzymes; AST, ALT, and T-Bil, Alb, and Glb (**a**–**e**) in treated rats with induced liver fibrosis compared to normal and CCl4-induced liver fibrosis groups. Data are expressed as average ± SD (*n* = 6). Where, ns indicates not significant, * *p* < 0.0332, ** *p* < 0.0021, *** *p* < 0.0002, **** *p* < 0.0001, versus control. ^#^ *p* < 0.0332, ^##^ *p* < 0.0021, ^###^ *p* < 0.0002, ^####^ *p* < 0.0001, versus CCl4 group.

The activities of alanine aminotransferases (ALTs), aspartate aminotransferases (ASTs), albumin (Alb), globulin (GLb), and total bilirubin (T. Bil) are among the liver’s activities that are tracked in the chronic liver disease rat groups treated with date palm seed coffee extract (PSCE), Arabica seed coffee extract (ACSE), and their corresponding nanoemulsions, including nanoemulsions of date palm seed coffee extract (NE-PSCE) and of Arabica seed coffee extract (NE-ACSE), as displayed in Figure 5. The control rat group showed significant decreases in AST, ALT, and T. Bil while showing significant increases in Alb and Glb compared with the group of non-treated diseased animals (CCl4). Rats with persistent liver damage treated with PSCE and ACSE had significantly lower levels of AST, ALT, and T. Bil than those in the group that was not treated (CCl4), while the levels of Alb and Glb were increased significantly in the PSCE and ACSE groups compared to the CCl4 group. In animals treated with nanoemulsions, including NE-PSCE and NE-ACSE, the levels of AST, ALT, and T. Bil decreased more than in either the PSCE or ACSE groups, whereas levels of Alb and Glb increased more than in the PSCE and ACSE groups. It can be observed that NE-ACSE and NE-PSCE were more effective in improving the levels of AST, ALT, T-Bil, Alb, and Glb for animals with chronic liver disease. In particular, NE-ACSE provided more effective treatment than other groups.

#### 3.5.3. Anti-Inflammatory Parameters

**Figure 6:**
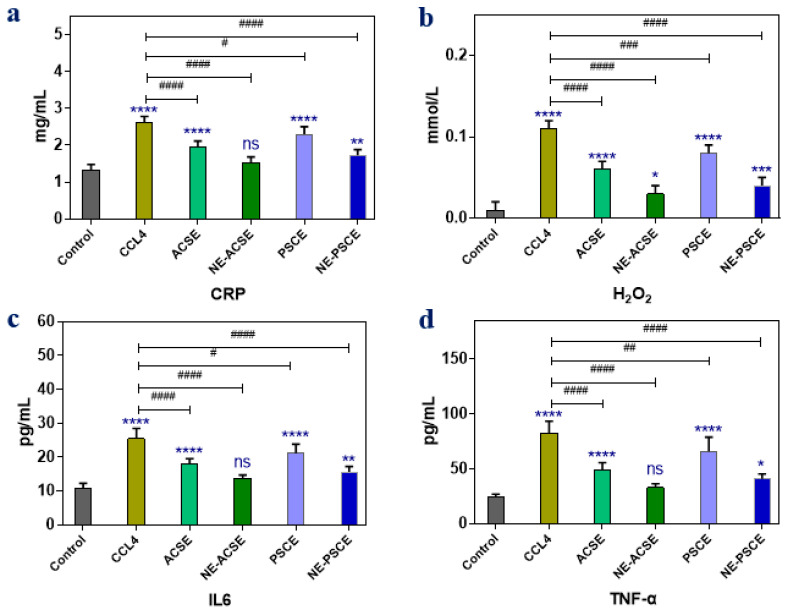
Effects of NE-PSCE and NE-ACSE on inflammatory parameters in treated rats with induced liver fibrosis compared to normal and CCl4-induced liver fibrosis groups with measurements of CRP (**a**), H_2_O_2_ (**b**), IL6 (**c**), and TNF-α (**d**). Data are expressed as average ± SD (*n* = 6). Where, ns indicates not significant, * *p* < 0.0332, ** *p* < 0.0021, *** *p* < 0.0002, **** *p* < 0.0001, versus control. ^#^ *p* < 0.0332, ^##^ *p* < 0.0021, ^###^ *p* < 0.0002, ^####^ *p* < 0.0001, versus CCl4 group.

Anti-inflammatory parameters including C-reactive protein (CRP), hydrogen peroxide (H_2_O_2_)_,_ interleukin-6 (IL6), and tumor necrosis factor alpha (TNF-α) were recorded to show the effects of extracts in normal forms or nanoemulsion forms on the levels of such inflammatory parameters, as displayed in Figure 6. It can be observed that the inflammatory markers CRP, H_2_O_2,_ IL6, and TNF-α were increased in the diseased liver group compared to control group, whereas the administration of extracts as ACSE and PSCE could minimize the levels of such parameters compared to those of the non-treated diseased liver group. In addition, the reduction in inflammatory markers was clearly observed when animals were treated with the nanoemulsion forms of the extracts, NE-PSCE and NE-ACSE. In accordance with the above results, NE-ACSE exhibited lower levels of the inflammatory parameters, as in the control.

#### 3.5.4. Liver Weight, Relative Liver Weight, Weight Gain, and Food Intake

**Figure 7:**
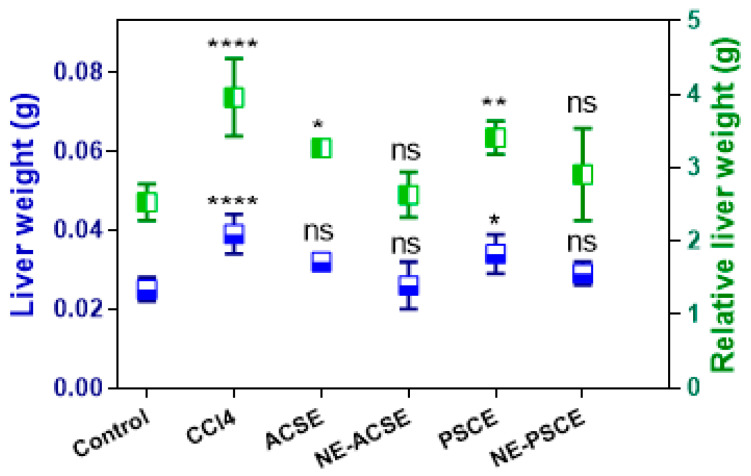
Effects of NE-PSCE and NE-ACSE on liver weight and relative liver weight in treated rats with induced liver fibrosis compared to the normal and CCl4-induced liver fibrosis groups. Data are expressed as average ± SD (*n* = 6). Where, ns indicates not significant, * *p* < 0.0332, ** *p* < 0.0021, **** *p* < 0.0001, versus control.

Liver weight and relative liver weight were recorded, as can be observed in Figure 7. According to the obtained results, the liver weight increased upon giving CCl4 to the diseased animal group with chronic liver disease in comparison with the control group. This was obvious in terms of the relative liver weight as well. As a result of treatment with extracts of both PSCE and ACSE, both liver weight and relative weight were decreased compared to the diseased CCl4 group. In addition, these decreases in the liver weight and relative liver weight were more obvious in diseased animals treated with NE-PSCE and NE-ACSE compared to the diseased non-treated group. Consistent with above results, NE-ACSE provided a more prominent effect.

The effects of ACSE, PSCE, and their nanoemulsion forms NE-ACSE and NE-PSCE on body weight gain and food intake in the chronic liver disease rat groups were determined as exhibited in Table 3. The effects of chronic liver disease on the diseased non-treated animals were negative in terms of health, feeding intake, and consequently weight gain, and such parameters were decreased significantly compared to the control group. On the other hand, weight gain and food intake were increased upon provision of the diseased animals with extracts of ACSE and PSCE. Moreover, the nanoemulsions of NE-ACSE and NE-PSCE could significantly improve weight gain and food intake compared to the diseased non-treated group. The nanoemulsions were more effective than the free extracts. Similar to the above results, NE-ACSE improved the status of the diseased animals, making it close to that of the control group.

#### 3.5.5. Histopathological Analysis

**Figure 8:**
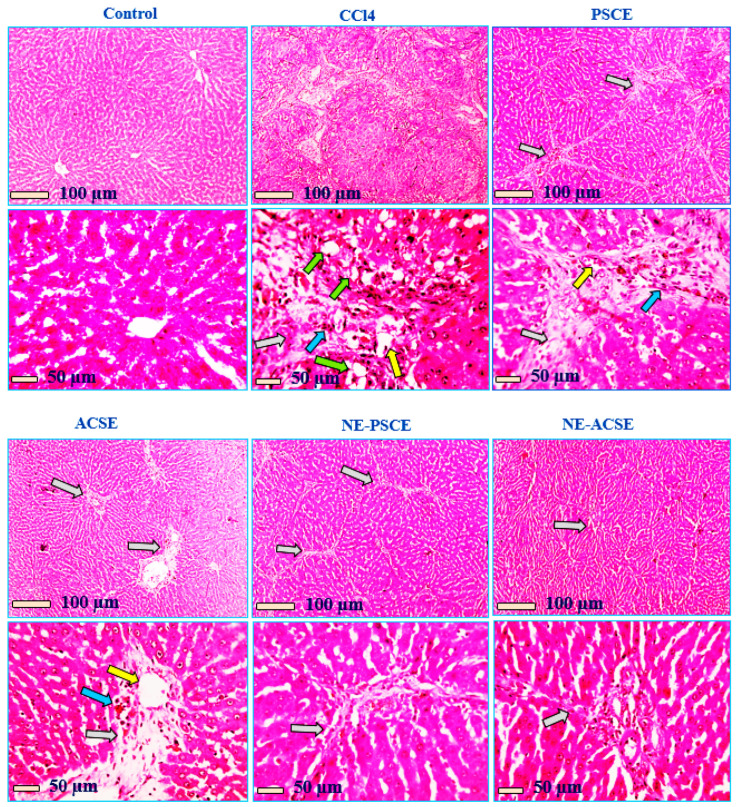
Histopathological analysis of rat model with induced liver fibrosis treated with PSCE, ACSE, and their nanoemulsions, NE-ACSE and NE-PSCE.

Histopathological analysis was performed for all groups—control, CCl4, PSCE, ACSE, NE-ACSE, and NE-PSCE—as can be observed in Figure 8. Under a microscope, hepatic slices stained with HE reveal a regular configuration of hepatic cords around central veins, together with normal portal regions and sinusoids in the normal control group. Hepatic sections from the positive group that received CCL4 show marked distension of portal areas due to fibrosis, leukocyte cell and hemosiderin-laden macrophage infiltration (blue arrow), congested blood vessels (yellow arrow), and thick anastomosing fibrous tissue extension from portal areas (grey arrow). Green arrows indicate ballooning degeneration in hepatocytes. Hepatic slices from the PSCE-treated group exhibit reduced portal area distension (grey arrow), a small infiltration of leukocyte cells and hemosiderin-loaded macrophages (blue arrow), less congested blood vessels (yellow arrows), and a long, thin fibrous tissue extension that anastomoses from the portal areas. Hepatic sections from the ACSE-treated group show greatly decreased distension of portal areas (grey arrows), fewer leukocytic cell infiltrations (blue arrows), few dilated blood vessels (yellow arrow), and few short non-anastomosing fibrous tissue extensions from portal areas. Hepatic sections from the NE-PSCE-treated group show few very short and very thin non-anastomosing fibrous tissue extensions from portal areas (blue arrows). Hepatic sections from the NE-ACSE-treated group show very few short and very thin non-anastomosing fibrous tissue extensions from portal areas (grey arrow).

## 4. Discussion

Regular coffee’s beneficial components have led to its classification as a functional food [62,63,64]. Particularly in wealthy nations, coffee is commonly consumed. Coffees are classified into broad categories according to how they are roasted or processed, which has an impact on the finished product’s chemical makeup. There are thus various varieties, including filtered and unfiltered coffee, as well as brewed, expresso, infused, instant, boiling, and decaffeinated coffee [65].

*Coffea canephora (robusta)* and *Coffea arabica* (*C. arabica*) are regarded as the two main species of coffee consumed globally. *C. arabica* is grown extensively, accounting for 70% of the world’s coffee production. One possible explanation for *C. arabica*’s exceptional quality is its organoleptic characteristics [66]. In Yunnan Province, China, *C. arabica* is widely used as a plant with significant medicinal value. Many of the chemical components of coffee have been separated thanks to advancements in contemporary technology and experimental methodologies. These components comprise sterols, taste compounds, alkaloids, flavonoids, terpenes, phenolic acids and their derivatives, and other substances. Numerous pharmacological actions, including those that are antioxidative, anti-inflammatory, anticancer, antidiabetic, liver-protecting, and neuroprotective, are a result of the chemical variety of its constituents. Caffeine, as a main constituent of *C. arabica*, was reported for its antioxidant properties, and in a wide range of dosages, caffeine is a safe xanthine alkaloid for human consumption [67]. The Food and Drug Administration classified caffeine as safe because the average adult’s hazardous amount is greater than 10 g [68]. Additionally, it has been reported that caffeine reduces oxidative stress, which may lessen liver damage and enhance neurological symptoms in rat models of hepatic encephalopathy [69]. Moreover, previous studies demonstrated that caffeine can partially prevent alcoholic liver fibrosis in rats, a condition that is assumed to be mediated by the cAMP-PKA-CREB signaling pathway [70,71]. It should be noted that the primary goal of the research is to identify the chemicals in *C. arabica* [72]. *C. arabica* beans are medium-roasted to produce Arabian coffee. It is a well-liked beverage in most Middle Eastern Arab nations [73]. Natural active substances found in abundance in *C. arabica* include sucrose, trigonelline, and chlorogenic acid [74]. *C. arabica* provides several pharmacological properties such as anti-inflammatory [75], antimicrobial [76], anticancer [26,77,78], and antioxidant properties [79]. Coffee attracts attention due to its significant application in clinical trials, in particular in inhibition of and protection against both alcoholic and non-alcoholic liver cirrhosis [80,81,82].

Date seed extract shows health benefits due to its antioxidant bioactive compounds including flavonoids, phenolic compounds, and vitamins, which have the capability to scavenge free radicals [83] and provide protection from hepatorenal toxicity [84]. GC/MS was used to examine Al-Baha date palm kernel (AB-DPK) extract. Antioxidants like 1-Trilinolein and (Z,Z) 3Dioctadecenoyl Glycerol as well as anti-tumor, anti-inflammatory, and antiviral medicines like botulin were among the compounds discovered in the investigation [85,86]. Extracts of the flesh and pits of dates were evaluated to ameliorate CCl4-induced hepatotoxicity in a rat model, and the extracts displayed significant hepatoprotective activity for animals that received the date extracts [87]. In a different study, when thioacetamide-treated animals were compared to control rats, there were notable increases in biochemical indicators of liver injury, such as ALT, AST, ALP, and total bilirubin, and a decrease in albumin levels. In addition, there was a substantial decrease in liver enzymes and an increase in serum albumin (*p* < 0.05) after treatment with extracts from palm dates. The hepatoprotective properties of palm date extracts may be due to their concentration of quercetin, which possesses potent antioxidant properties [88]. The power *of P. dactylifera* seed extract to scavenge DPPH could be attributed to the high antioxidant capacity of the extract contents. It was demonstrated that the antioxidant properties of date palms could have emerged from available compounds such as selenium, vitamin E, ascorbic acid, flavonoids, tannins, and other phenolic compounds [89,90].

The levels of intracellular enzymes, including AST and ALT as well as T. Bil, are biomarkers and significantly sensitive to hepatic injuries [91]. As a result of leakage of the cell membrane associated with a loss of membrane integrity, the activities of ALT and AST are increased; meanwhile, the impairment in the biliary function of the liver and the associated damages to hepatic tissues result in an increase in the level of T-Bil [92,93]. ALT, AST, and T-Bil serum levels in rats exposed to PSCE or ACSE were reduced compared to those in the chronic liver disease group in this study. Such results attest to the safety profile and protective therapy of both ACSE and PSCE in restoring liver functions. In a previous study, methanolic and aqueous extracts of *Phoenix dactylifera* were demonstrated as protective products against oxidative and hepatic injuries induced by paracetamol. According to the study, the extracts worked through blocking the oxidative stress caused by the paracetamol, thus ameliorating the induced hepatocellular injury and elevated serum ALT and AST [94]. In another study which used aqueous Ajwa date fruit extract as an anticancer agent to protect against and treat hepatocellular carcinoma induced using diethylnitrosamine in a rat model, the extract could improve liver functions due to its antioxidant activity, and the levels of ALT and AST were reduced [95]. As a result of the capability to improve kidney and liver functions that emerges from their potent antioxidant properties, there is the possibility of using date seed extracts as anticancer agents against colorectal and hepatocellular carcinoma cell lines, but further investigations are still required [96].

According to the obtained results of this study, the *Coffea arabica* and *Phoenix dactylifera* nanoemulsions NE-ACSE and NE-PSCE provided more significant therapeutic effects against CCl4-induced chronic liver disease in a rat model compared to the free extracts. These results were obvious due to reductions in liver intracellular enzymes such as AST, ALT, and T. Bil. Also, the inflammatory parameters, such as IL6, TNF alpha, CRP, and H_2_O_2_, were significantly decreased for diseased animals that received either nanoemulsion, NE-ACSE or NE-PSCE, compared to the CCl4 group and those provided with free extracts. Also, antioxidant enzymes such as SOD, CAT, GSH, and GPx were improved significantly. Furthermore, the health status parameters, including food intake, weight gain, and food efficiency rate, were improved more by using nanoemulsions than by using free extracts. Such results were confirmed and were consistent, as displayed in histopathological examinations, where the nanoemulsions NE-ACSE and NE-PSCE could decrease the cellular infiltrations and damages more than the free extracts. The obtained results, which indicate the high efficiency of nanoemulsions of NE-ACSE or NE-PSCE as protective agents in induced chronic liver disease, could be attributed to the high stability of the extracts when they are nanoemulsified, and the slow and sustained release of the extracts over a longer period than that of the free extracts. Because of the special qualities of the involved nanosized droplets, such as large surface area, nanoemulsions are being studied in the fields of food and healthcare [97]. Furthermore, texture, stability, absorption, absorbability, fortification, and bioavailability will all be improved by the extracts’ active components in nanoemulsified latex [98,99,100]. Additionally, nanoemulsions can protect natural components from degradation, thereby extending their shelf life and preserving their efficacy. Bioactive substances can eventually deteriorate due to direct exposure to external influences including heat, light, and oxygen, and this is prevented by encapsulation. Furthermore, nanoemulsions can improve the targeted delivery of the natural components of PSCE and ACSE to the targeted affected organ/tissue within the body.

## 5. Conclusions

The present study reported on nanoformulations of ACSE and PSCE with the aid of oleic acid and the Tween 80 system. To the best of our knowledge, the influence of the developed nanoemulsions NE-PSCE and NE-ACSE on induced liver fibrosis in a rat model was investigated herein for the first time. The major findings reveal that both NE-ACSE and NE-PSCE were allocated in nanoscales with negative ζ-potentials. The antioxidant activity was determined, and ACS and PSC extracts and their nanoemulsions displayed antioxidant properties. In a rat model with liver fibrosis induced via CCl4, the influence of both nanoemulsions was demonstrated in the aspects of inflammatory parameters, liver functions, antioxidant enzymes, and histopathological analysis. In comparison to normal extracts of ACSE and PSCE, the nanoemulsions NE-ACSE and NE-PSCE provided significant improvements in antioxidant enzymes and decreases in inflammatory parameters, and the liver functions of ALT and AST were decreased to be close to those of the control group. It can be concluded that nanoemulsions of ACSE and PSCE provide a new avenue as a therapeutic approach to liver diseases, and further studies are encouraged to obtain maximum efficiency of treatments via the combination of both extracts in nanoforms.

## Figures and Tables

**Table 1:** GC-MS of ACS extract.

S. No.	RT	Name of the Compound	Molecular Formula	M.W.	Area %
1	9.33	Trimethylsilyl)oxy]phenol	C9H14O2Si	182	0.67
2	11.36	Niacin, TMS derivative	C9H13NO2Si	195	0.78
3	11.65	Methoxy-4-vinylphenol	C9H10O2	150	0.71
4	14.01	10,11-Dimethylbicyclo [6.3.0]undec-1(8)-en-9-one	C13H20O	192	0.68
5	14.78	2,6-Dicarbomethoxy-4-chloroterahydropyran	C13H20O	192	0.58
6	16.13	2,2-Dimethyl-5-[2-(2-trimethylsilylethoxymethoxy)-propyl]-[1,3]dioxolane-4-carboxaldehyde	C15H30O5Si	318	1.27
7	16.95	Triethylene glycol, 2TMS derivative	C12H30O4Si2	294	0.63
8	17.34	6-D1-androst-5-en-3á-ol	C19H31DO	227	1.43
9	17.99	2,2,18,18-Tetramethyl-3,6,10,13,17-pentaoxa-2,18-disilaneonadecane	C16H38O5Si2	366	0.37
10	18.13	2,2,18,18-Tetramethyl-3,6,10,13,17-pentaoxa-2,18-disilaneonadecane	C16H38O5Si2	366	0.49
11	18.23	Tripropylene glycol monomethyl ether, TMS derivative	C13H30O4Si	278	0.34
12	18.63	à-L-Galactopyranoside, methyl 6-deoxy-2-O-(trimethylsilyl)-, cyclic methylboronate	C11H23BO5Si	274	0.97
13	20.30	Dodecanoic acid, TMS derivative	C15H32O2Si	72	0.54
14	21.60	á-D-Galactopyranoside, methyl 2,6-bis-O-(trimethylsilyl)-, cyclic methylboronate	C14H31BO6Si2	362	0.56
15	23.22	1H-Purine-2,6-dione, 3,7-dihydro-1,3,7-trimethyl-	C8H10N4O2	194	56.36
16	24.43	Myristic acid, TMS derivative	C17H36O2Si	300	0.73
17	25.68	Pentadecanoic acid, 14-methyl-, methyl ester	C17H34O2	270	0.32
18	26.47	Hexadecanoic acid	C16H32O2	256	2.53
19	28.22	Palmitic Acid, TMS derivative	C19H40O2Si	328	10.81
20	29.44	9,12-Octadecadienoic acid (Z,Z)-	C18H32O2	280	1.29
21	29.62	trans-13-Octadecenoic acid	C18H34O2	282	1.65
22	30.08	Octadecanoic acid	C18H36O2	284	0.41
23	30.99	9,12-Octadecadienoic acid (Z,Z)-, TMS derivative	C21H40O2Si	352	0.94
24	31.14	9-Octadecenoic acid, (E)-, TMS derivative	C21H42O2Si	345	1.49
25	31.67	Stearic acid, TMS derivative	C21H44O2Si	356	0.63
26	34.08	Creatindial	C20H24O2	296	0.81
27	34.53	5,16,20-Pregnatriene-3beta,20-diol diacetate	C25H34O4	389	3.23
28	34.78	(20R)-18,20-Epoxypregn-5-en-3á-yl acetate	C21H30O	289	0.71
29	35.14	9-Anthracenol, 1,4,8-trimethoxy-2-methyl-	C18H18O4	298	3.37
30	35.63	Aarda-5,20(22)-dienolide, 3,14,19-trihydroxy-, (3á)-	C23H32O5	388	1.05
31	36.20	5-Iodo-6-formyl-3,4-dimethoxy-2,2’-bipyridine	C13H11IN2O3	370	1.10
32	40.44	Aucubin, hexakis(trimethylsilyl) ether	C33H70O9Si6	778	0.33
33	44.22	Stigmasta-5,22-dien-3-ol	C29H48O	412	0.58
34	44.74	á-Sitosterol	C29H50O	414	0.93
35	45.23	9,12-Octadecadienoic acid (z,z)-, 2,3-bis[(trimethylsilyl)oxy]propyl ester	C27H54O4Si2	498	0.69

**Table 2:** GC-MS of PSC extract.

S. No.	RT	Name of the Compound	Molecular Formula	M.W.	Area %
1	9.32	à-Phenylbenzenemethyl 4-nitrobenzoateate	C20H15NO4	333	1.03
2	10.62	Diethylene glycol, 2TMS derivative	C10H26O3Si2	250	0.76
3	13.16	Tetraethylene glycol, TMS derivative	C11H26O5Si	266	5.23
4	14.84	Tripropylene glycol monomethyl ether, TMS derivative	C13H30O4Si	278	0.80
5	14.94	Tripropylene glycol monomethyl ether, TMS derivative	C13H30O4Si	278	1.30
6	15.03	1-(1-Butoxy-2-propoxy)-2-propanol, TMS derivative	C13H30O3Si	262	1.44
7	15.12	1-(1-Butoxy-2-propoxy)-2-propanol, TMS derivative	C13H30O3Si	262	0.97
8	16.96	Triethylene glycol, 2TMS derivative	C12H30O4Si2	294	9.98
9	17.75	Tripropylene glycol monomethyl ether, TMS derivative	C13H30O4Si	278	1.20
10	17.99	Tripropylene glycol monomethyl ether, TMS derivative	C13H30O4Si		1.56
11	18.13	Tripropylene glycol mono-n-butyl ether, TMS derivative	C16H36O4Si	320	1.99
12	18.24	Tripropylene glycol monomethyl ether, TMS derivative	C13H30O4Si	278	2.23
13	20.30	Dodecanoic acid, TMS derivative	C15H32O2Si	272	4.37
14	21.59	Cyclopentanetridecanoic acid, methyl ester	C19H36O2	296	0.68
15	24.42	Myristic acid, TMS derivative	C17H36O2Si	300	3.62
16	25.68	Pentadecanoic acid, 14-methyl-, methyl ester	C17H34O2	270	0.67
17	26.41	Hexadecanoic acid	C16H32O2	256	0.62
18	26.49	d-Galactose, 2,3,4,5,6-pentakis-O-(trimethylsilyl)-, o-methyloxyme, (1Z)-	C22H55NO6Si5	569	0.67
19	28.22	Palmitic Acid, TMS derivative	C19H40O2Si	328	15.32
20	28.86	10-Octadecenoic acid, methyl ester	C19H36O2	296	1.27
21	29.61	cis-13-Octadecenoic acid	C18H34O2	282	2.87
22	29.74	Benzoxepino [5,4-b]pyridine-3-carbonitrile, 5,6-dihydro-2-methyl-4-(methylthio)-	C16H14N2OS	282	0.86
23	29.90	9-octadecenamide	C18H35NO	281	0.48
24	30.91	Decaethylene glycol, 2TMS derivative	C26H58O11Si2	602	0.40
25	30.99	Linoelaidic acid, trimethylsilyl ester	C21H40O2Si	352	2.03
26	31.16	Oleic Acid, (Z)-, TMS derivative	C21H42O2Si	354	12.63
27	31.67	Stearic acid, TMS derivative	C21H44O2Si	356	1.90
28	32.08	Glycidyl palmitate	C19H36O3	312	0.58
29	32.84	9-octadecenamide	C18H35NO	281	1.16
30	34.87	Glycidyl oleate	C21H38O3	338	1.10
31	35.86	Diisooctyl phthalate	C24H38O4	390	1.22
32	36.57	1,3-Dipalmitin, TMS derivative	C38H76O5Si	640	1.02
33	37.94	9-Octadecenoic acid (Z)-, 2-hydroxy-1-(hydroxymethyl)ethyl ester	C21H40O4	356	1.30
34	38.98	9-Octadecenoic acid (z)-, 2-[(trimethylsilyl)oxy]-1-[[(trimethylsilyl)oxy]methyl]ethyl ester	C27H56O4Si2	500	0.88
35	39.10	9-Octadecenoic acid (z)-, 2-[(trimethylsilyl)oxy]-1-[[(trimethylsilyl)oxy]methyl]ethyl ester	C27H56O4Si2	500	2.32
36	40.45	D-(+)-Trehalose, octakis(trimethylsilyl) ether	C36H86O11Si8	918	1.52
37	43.20	9,12,15-Octadecatrienoic acid, 2,3-bis[(trimethylsilyl)oxy]propyl ester, (z,z,z)-	C27H52O4Si2	496	1.14
38	43.43	2-(dodecanoyloxy)-1-(hydroxymethyl)ethyl laurate #	C27H52O5	456	0.88
39	43.92	Ethyl iso-allocholate	C26H44O5	436	0.69
40	44.15	Lup-20(29)-en-3-one	C30H48O	424	1.09
41	44.75	ç-Sitosterol	C29H50O	414	5.87
42	44.87	Cholest-5-en-3-ol, 24-propylidene-, (3á)-	C30H50O	426	1.26

**Table 3:** Effects of NE-PSCE and NE-ACSE on weight gain (%) and food intake in treated rats with induced liver fibrosis compared to the normal and CCl4-induced liver fibrosis groups. Data are expressed as average ± SD (*n* = 6). Where, ns indicates not significant, ** *p* < 0.0021, *** *p* < 0.0002, **** *p* < 0.0001, versus control.

Group	Weight Gain %	Food Intake (g)
Control	30.29 ± 6.5	24.48 ± 3.71
CCl4	17.48 ± 5.24 ***	16.32 ± 0.32 ****
ACSE	24.65 ± 3.92 ^ns^	24.40 ± 0.49 ^ns^
NE-ACSE	29.07 ± 4.37 ^ns^	25.89 ± 0.34 ^ns^
PSCE	24.21 ± 3.36 ^ns^	20.39 ± 0.84 **
NE-PSCE	25.63 ± 1.31 ^ns^	24.49 ± 0.21 ^ns^

## Data Availability

Data is contained within the article and Supplementary Materials.

## Supplementary Materials

The following supporting information can be downloaded at: https://www.mdpi.com/article/10.3390/antiox13030355/s1. Figure S1. Compounds Identified in the ACS extract using GC-MS; Figure S2. Compounds Identified in the PSC extract using GC-MS; Figure S3. Nanoemulsion of (a) NE-ACSE and (b) NE-PSCE; Figure S4. ζ-potential (a, b) of the developed NE-ACSE and NE-PSCE, respectively.
