# Supplementary material for: Nanoemulsions of Phoenix dactylifera L. (Decaffeinated) and Coffea arabica L. Extracts as a Novel Approach for the Treatment of Carbon Tetrachloride-Mediated Liver Fibrosis
Source: Antioxidants (Basel). 2024 Mar 16;13(3):355. doi: 10.3390/antiox13030355 (PMC10968094; doi:10.3390/antiox13030355)
Supplement: Supplementary file 1 [file antioxidants-13-00355-s001.zip › antioxidants-2882637-supplementary.pdf]

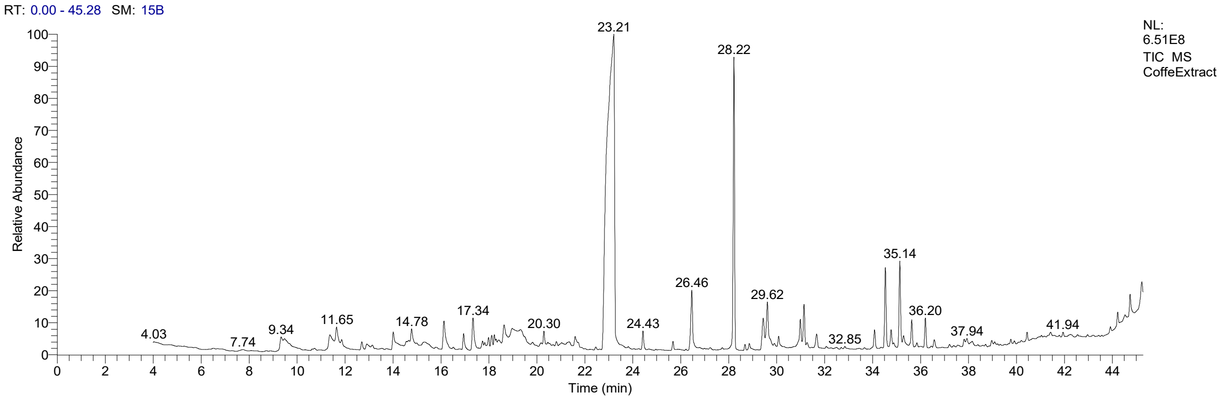

Figure S1. Compounds Identified in the ACS extract using GC-MS

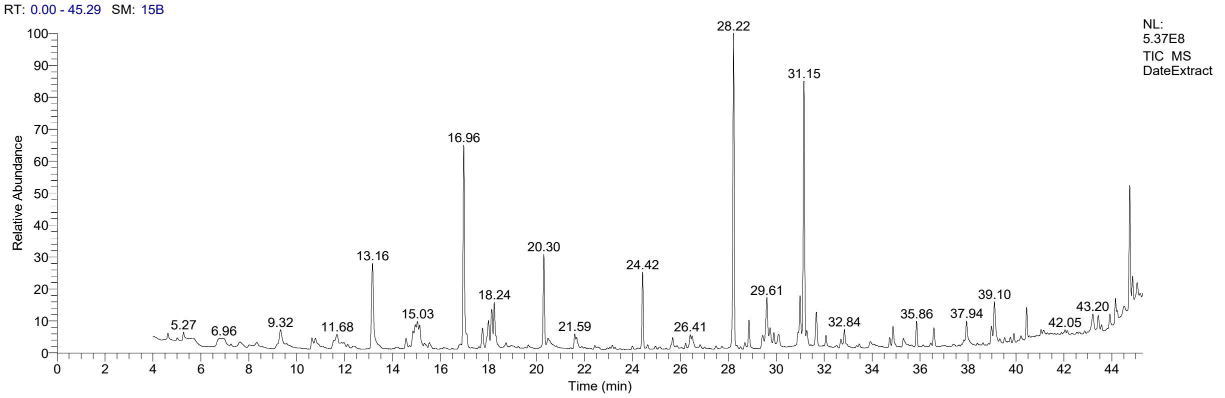

Figure S2. Compounds Identified in the PSC extract using GC-MS

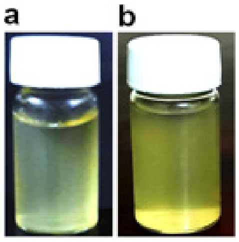

Figure S3. Nanoemulsion of (a) NE-ACSE and (b) NE-PSCE

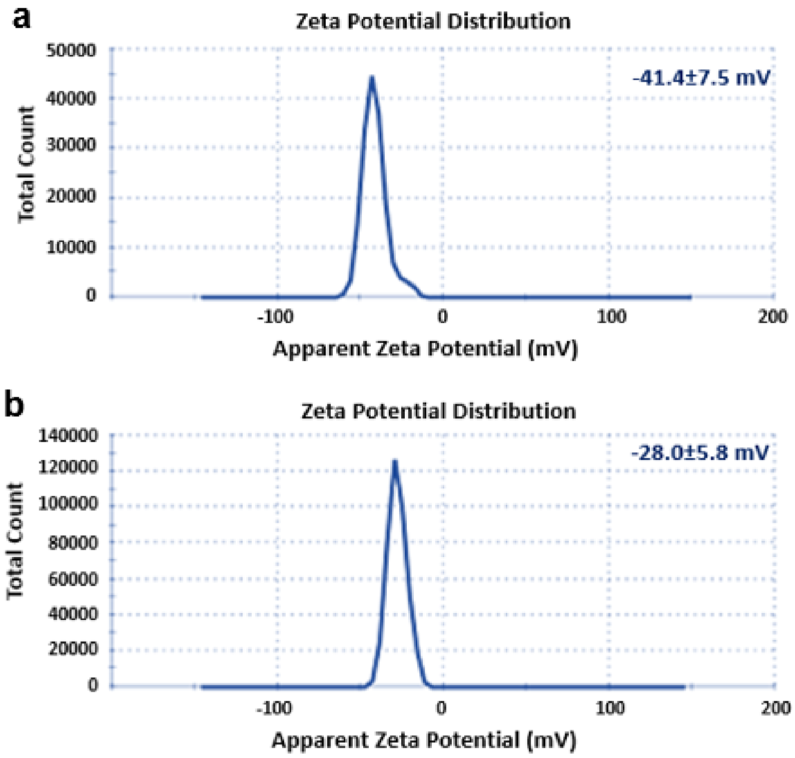

Figure S4. ζ-potential (a, b) of the developed NE-ACSE and NE-PSCE, respectively.
